# The Contribution of Intersectoral Healthcare Centres with an Extended Outpatient Care Model to Improve Regional Care-Structures—A Qualitative Study

**DOI:** 10.3390/ijerph20075365

**Published:** 2023-04-03

**Authors:** Heidrun Sturm, Florian Kaiser, Philipp Leibinger, Edgar Drechsel-Grau, Stefanie Joos, Andreas Schmid

**Affiliations:** 1 Institute of General Practice and Interprofessional Care, University Hospital Tübingen, 72076 Tübingen, Germany; 2 Oberender AG, Wahnfriedstraße 3, 95440 Bayreuth, Germany; 3 Management im Gesundheitswesen, Rechts-und Wirtschaftswissenschaftliche Fakultät, Universität Bayreuth, Universitätsstraße 30, 95447 Bayreuth, Germany

**Keywords:** integrated care, intersectoral care, primary care, primary care centers, short-term care

## Abstract

Many smaller hospitals in Germany are currently threatened with closure due to economic reasons and politically derived centralization. In some—especially rural areas—this may result in a lack of accessible local care structures. At the same time, patients are unnecessarily admitted to hospitals due to insufficient primary care structures and healthcare coordination. Intersectoral health centers (IHC), as new intermediary structures, may offer round-the-clock monitoring (Extended Outpatient Care, EOC), with fewer infrastructure needs than hospitals and, thus, could offer a sustainable solution. In an iterative process, 30 expert interviews (with physicians, nurses and other healthcare experts) formed the basis for the derivation of diagnostic groups, relevant related patient characteristics and scenarios, as well as structural preconditions necessary for safe care in the setting of the new model of IHC/EOC. Additionally, three workshops within the multidisciplinary research team (including healthcare services researchers, GPs, and health economists) were performed. Inductive categories on disease-, case-, sociodemographic- and infrastructure-related criteria were derived following thematic analysis. Due to the expert interviews, general practice equipment plus continuous monitoring beds should form the basic infrastructure for EOCs, which should be adjusted to local needs and infrastructure demands. GPs could be aided through (electronic) support by other specialists. IHC, as a physician-led facility, should rely on experienced nurses to allow for 24-h services and to support integrated team-based primary care with GPs. Alongside nurses, case managers, therapists and social workers can be included in the structure, allowing for improved integration of (primary) care services. In order to sustain low-threshold, local access to care, especially in rural areas, IHC with extended monitoring and integration of coordinative support, emerged as a promising solution that could solve many common patient needs without the need for hospital-based inpatient care.

## 1. Introduction

This paper focuses on a care model conceptualized for Germany that was elaborated in a scientific opinion for the National Association of Statutory Health Insurance Physicians [1]. It introduces a new intermediate care level, offering extended outpatient care (EOC) in the setting of so-called intersectoral health centers (IHC). It features a small inpatient ward to observe and treat patients over a period of a couple days without requiring a complete hospital infrastructure. Rather, IHCs are building on services and structures provided by regular GP practices, supplemented by 24/7 experienced nurses supplemented by physicians on-call, as detailed by Schmid et al., 2018 [2].

Internationally, a range of different factors drive the interest in alternatives to hospital care. Firstly, rural regions need solutions for access to higher acuity patient care to supplement primary care; hospitals are difficult to sustain in remote and sparsely populated areas. Thus, in some countries’ health care delivery, structures to bridge primary and hospital care were established. For example, in Finland in 2015, 226 municipal (primary) health centers featured “inpatient hospital-type wards” [3] (p. 103). In those—especially in rural areas—physicians or nurses are present intermittently due to limitations in staffing, but also, about 50% of acute cases do not require physicians’ intervention and can be dealt with by a nurse [4,5]. In the US, so-called Micro-Hospitals are created that typically are licensed for a minimum of 8 beds, seeing 25 to 80 emergency patients a day [6,7]. However, internationally, such facilities are not typical.

Secondly, demographic changes require coordinated and continuous care for the growing elderly population with multiple chronic conditions [8,9,10,11]. Therefore, comprehensive primary care, defined by comprehensive coordinated care and patient-centeredness, is central for coping with increasingly complex care needs in the future [12].

The German healthcare system is characterized by pronounced sectoral divisions leading to disruption of care, especially between inpatient and outpatient care or rehabilitation. General practitioners (GPs), as well as medical specialists, mostly work in private practices, where smaller units such as solo or very small group practices still prevail [13], although their number is declining. In addition, there are separate funding systems and budgets for the hospital sector and outpatient sector.

A German study found that out of a set of defined so-called “ambulatory care-sensitive conditions” on average, 75% of a given diagnosis could be handled in the outpatient setting, if outpatient care structures were improved [14]. For quality reasons, but also because of reduced infrastructure requirements and costs, a stated aim of healthcare planners for many years has been to avoid (unnecessary) hospitalizations by strengthening outpatient care. Little has changed, however.

On the contrary, in rural Germany, outpatient (primary) care providers are scarce as the present “baby-boomer” generation of physicians is retiring; over 35% of GPs are older than 60 years, and 15% older than 65 [15]. The younger generation of German doctors prefers being employed rather than self-employed, embracing defined working hours in favor of autonomy, and they prefer interprofessional teams to one-doctor practices [16,17]. In addition, there is a shortage of pharmacists, therapists, nurses and other health professionals [18].

At the same time, health policy strongly favors the closure of small hospital sites (which traditionally had been installed in almost every county based on political rather than medical needs) and centralization. Thus, small hospitals are struggling financially and are short-staffed [19]. Such small hospitals do not typically cater to complex inpatient treatments, and their patients do not need complete hospital infrastructures; however, without some form of replacement, barriers to care are likely. In December 2022, the *government commission for modern and needs-based hospital care*, newly installed by the federal ministry of health, issued an advisory statement to introduce a new level of German hospital typologies. This new level “1i” represents nurse-led facilities very similar to the aforementioned IHC, thus giving new dynamics to the discussion [20].

Taken together, care needs to be organized in a comprehensive manner, utilizing all available capacities and competencies to their maximum. The Federal Advisory Council on the Assessment of Developments in the Health Care System has repeatedly asked for a more integrated, needs-oriented and population-based care, including better coordination [21]. Against this background, calls for interprofessional primary care centers to overcome the challenges are increasing and come from various stakeholders in Germany. However, the model has remained in a vague conceptual stage, as the same stakeholders and legislature have not yet found common ground on the regulatory setup for such facilities.

Primary healthcare centers (without EOC) that offer interprofessional and integrated care and provide a first point of contact to patients are implemented and positively evaluated in many countries [22,23,24,25]. In recent years, reviews and studies have been devoted to the effects and benefits of integrated care and care with an expanded “skill mix” in various health care systems [26,27,28,29,30]. It has been shown that good task distribution in interprofessional teams can increase care capacity while at least maintaining, if not improving, quality [31]. Especially in life-style-related conditions, interprofessional teams can help to improve prevention, adherence to therapy and avoid disruption of care [32]. For Germany, Arnold et al. showed the importance of continuity of care in cardiology [33]. Concepts similar to EOC have been implemented and partly evaluated in some countries with comparable healthcare organizations. In Switzerland, for instance, being treated in such a facility resulted in increased patient satisfaction along with lower costs, while there were no indications of increased complications or re-admissions [34]. Even in Germany, single pilot projects were able to demonstrate that hospitalizations could be reduced [35] and patient satisfaction improved [36]. However, those German pilots were still based on a regular hospital setup. A recent overview described German pilot projects and their organizational setting, however, without effect analysis [37].

In this contribution which is based on a conceptual study funded by the National Association of Statutory Health Insurance Physicians [1] and focuses on EOC provided in IHC, we hypothesize:

(a) that such facilities can offer comprehensive care close to home for conditions that are presently treated in hospitals. Therefore, IHCs are expected to free hospital beds for patients more in need and, at the same time, ensure access to higher-level care, especially in rural areas where small hospitals are increasingly difficult to sustain.

(b) IHC infrastructure needs will be considerably below those of a regular hospital without compromising the quality of care.

(c) IHCs with EOC offer an intermediate level of care that addresses patient needs better than present structures, especially the needs of the growing group of multimorbid geriatric patients.

Based on these hypotheses, with this qualitative study, we aim to define patient groups who can benefit from and be safely treated in the setting of EOC. Derived from this, services and related infrastructure needed to care for these patients will be defined in order to provide guidance for healthcare planners.

On a policy level, this should/may contribute to a better understanding of the scope as well as the potential benefit of such facilities. This, in turn, may reduce barriers to their implementation. Despite being conceptual, the study also contributes to the currently limited literature on such forms of care.

## 2. Materials and Methods

In an iterative process, expert interviews formed the basis for the derivation of diagnostic groups, relevant related patient characteristics as well as structural preconditions necessary for care in the setting of the new model of IHC/EOC. Additionally, three workshops within the multidisciplinary research team (including healthcare services researchers, GPs, and health economists) were performed. The steps in detail:

(1) To better understand the potential and challenges of IHC, in the first step, we conducted 30 semi-structured explorative expert interviews with the goal to detect as many suitable indications for EOC as possible without the risk of narrowing down to the lowest common denominator.
(2) In most cases, conditions as expressed in ICD codes as such, did not sufficiently clarify if a patient can be treated safely in the EOC environment. Therefore, to define suitability, criteria based on the disease and the patient’s social context were established. In addition, the perceived added value of the proposed interprofessional and integrated care structures, and the perceived benefits of being admitted short-term to EOC were derived.
(3) Subsequently, and again based on the interviews, procedures necessary for the treatment of the respective conditions were identified and, in a second step, grouped to derive a spectrum of procedures that should be offered in each center.
(4) The necessary expertise, as well as technical infrastructure for EOC, was derived based on these results along with regulatory requirements, clinical needs, etc.

Taken together, this describes the structural framework for the proposed healthcare structures. In a related workstream, based on the interview results, diagnoses and/or scenarios suitable for EOC (“EOC-sensitive conditions”) were listed, and ICD codes were assigned. For further reference, see [1].

As experts for the interviews, in addition to nurses, healthcare planners and consultants, a variety of medical specialists working in hospitals and private practices as well as in rural and urban areas were included (See Table 1). Specialty selection was supported by empirical data, i.e., most frequent diagnoses of inpatient cases without surgery and with low case severity scoring were derived from a standardized hospital dataset (§21 KHEntgG) and complemented by established ambulatory sensitive diagnoses [14] to be then grouped to rank the relevance of different specialties for EOC type care. Experts were contacted via phone or mail based on the regional network and snowball sampling.

The interviews focused on the following four key questions: 1. What are typical diagnoses or cases/constellations for whom the setting of EOC would be advantageous? 2. What kind of diagnostic and therapeutic procedure could be safely delivered in this setting? 3. Which infrastructural and personnel-related resources are needed for that? 4. Which chances and barriers do you see for this care structure?

Almost all interviews were conducted via Zoom. Prior to each interview, the EOC concept was presented to the interviewee, and the interview started with an open question, asking participants about their general perception of such care structures. If necessary, above-mentioned diagnoses served as examples to widen the discussion. Interviews were recorded and transcribed, and analyzed in the first step following thematic analysis [38].

## 3. Results

The subsequent results are based on the analysis of the 30 expert interviews. Ethics approval was obtained and is listed in the back matter of this article.

### 3.1. Decision Criteria for the Admission to EOC

Rather than focusing on distinct diagnoses, experts mainly provided clinical scenarios that they deemed to be safely manageable in an EOC setting (Table 2). Those scenarios could be grouped into (1) acute, potentially life-threatening conditions, (2) crisis intervention, (3) exacerbation of chronic disease states and (4) palliative/supportive care as well as (5) diagnostic or therapeutic procedures.

As a precondition for the safe care of acute diseases (especially in younger patients), it was requested to exclude the risk of complications that could not be handled in an EOC setting, thus heightening the importance of reliable diagnoses. Conditions that need regular monitoring and adjustments of medication or IV therapy can be handled in EOC. As compared with outpatient care, a wider spectrum of diagnostic as well as therapeutic procedures can be performed safely under EOC conditions, whilst avoiding hospital admission. Additionally, in phases where home care is not possible for a period of time, yet the threshold for admission to a full hospital infrastructure is not met, EOC offers interim solutions at the most appropriate level of care. In chronic patients, where the diagnosis is established, especially for exacerbation or therapy adjustments, EOC was considered a suitable care structure. This setting seems ideal for increased monitoring during medication adjustment or diagnostic tests, plus temporary support in palliative or demented patients.

Thus, the medical decision between outpatient care and EOC is mainly defined by the necessity of continuous monitoring, re-assessment and parenteral medication. However, the individual decision regarding the level of care appeared to depend on additional criteria (Table 3): disease-related criteria; individual case severity; sociodemographic criteria; and structure-related criteria. Thus, next to individual circumstances, the available infrastructure in a given center also defines the spectrum of diseases that can be safely treated in EOC.

### 3.2. Procedures and Services to Be Provided in an EOC

Derived from the interviews, the range of necessary procedures and services was defined. Procedures were grouped into: (1) *diagnostics* (divided into technical, consultant or lab-based), (2) *monitoring and assessments*, (3) *planning and coordination of therapy* and (4) *therapy itself* (invasive procedure, medication, nursing care).

In addition, it was distinguished between *basic services* that have to be provided in every EOC and *facultative services*, which are provided depending on the regional resources. If, for instance, additional medical specialists form part of the network (either by local integration in the center or affiliated with a wider network), the spectrum of procedures can be widened by providing “satellite surgeries” or (telehealth) consults/counseling. In turn, specialists can increase their range of procedures by taking advantage of this structure, especially if follow-up monitoring is required.

Some examples of procedures that can be performed by outpatient specialists but are safer within EOC structures if certain patient-related risk factors apply:

- Rheumatology/Orthopedics: Diagnostic joint punctures
- Gastroenterology: endoscopies, liver punctures, PEG placement and changes
- Diabetology: adjusting therapy of diabetic patients that are difficult to control
- Oncology: diagnostic bone marrow punctures (e.g., in case of neoplasia)
- Cardiology: transesophageal echo (TEE), cardioversion (electrical + medicinal)
- Neurology: EEG, handicapped patients
- Ophthalmology: eye pressure profiles, e.g., for glaucoma patients
- Surgery/Dermatology: Minor surgical procedures, skin biopsies
- Urology: Permanent catheter placement
- Dentistry/Oral surgery: dental interventions (under short anesthesia)
- Obstetrics: CTG monitoring, hyperemesis

### 3.3. Necessary Infrastructure and Expertise

In line with our initial supposition, the resulting necessary technical infrastructure and equipment for a basic EOC version is the equivalent of that found in a typical GP practice with an additional ward-like structure with beds to allow inpatient stays.

Infrastructure and procedures depend on each other and therefore have to be adjusted to the regional settings. Besides that, based on the procedures and therapies to be offered in the EOC, the necessary qualifications and experience of physicians and other staff were described by the experts. In addition to GPs and nurses, a wide range of physician specialists and other health professionals were considered to be of additional value in an interprofessional setting. In addition, the necessary qualification and competencies for each profession (such as geriatric or psychiatric expertise, patient education, wound management, etc.) were detailed, and the requirements of a functioning collaboration were specified. In addition, an interprofessional and team-based working approach was emphasized repeatedly.

The following key issues concerning cooperation and structure were raised in comments by the interviewees:

- A functioning team consisting of GPs, nurses and practice managers serves as a base
- Importance of a geriatric team is emphasized consisting of GPs, nurses, therapists
- New roles within teams must be discussed and defined; however, roles and responsibilities in patient care are not yet fully defined
- Home visits by nurses or practice organizers can be supported by telemedicine
- Qualification of practice managers requires a medical background (e.g., Advanced Practice Nurses) and should include experience in interprofessional and complex care settings
- Physicians in the wider network should do mutual internships to improve mutual understanding,
- Physicians in training should be included
- The creation of redundant structures has to be avoided

## 4. Discussion

The expert-based explorative study provides multi-perspective information to define distinct patient groups that can vastly profit from an intermediate structure such as EOC. Alongside diagnostic groups, additional criteria related to individual patients and regional structures could be developed to support the decision of care within an EOC is appropriate.

Whilst the fundamental is general practice, integrated, and therefore coordinated care, forms the principle of care in EOC settings. Participating experts assumed that intersectoral care centers that offer interprofessional and intersectoral care with overnight stays can provide a number of advantages: Interprofessional services were seen to be especially useful (e.g., pain management, geriatric trauma, geriatric prehab or diagnostics that require various specialties). The formation of regional care networks with the IHC as a hub was seen as a further advantage. However, experts also highlighted concerns such as the potential creation of redundant structures and uncertainty with regard to roles and responsibilities in the care of patients.

### 4.1. Patient-Oriented Care

For patients, it was considered a major advantage that short-term intensified medical needs can be solved close to home instead of in larger and usually more distant hospitals [7]. Local care providers can be continuously included in the care process, avoiding the anonymity and stress that sometimes comes with larger and more distant hospitals, especially for elderly patients. Furthermore, local treatment reduces the risk of information loss or interruption of accompanying therapies, e.g., physiotherapy. Especially geriatric patients can profit from the presence of family members; it was even suggested to offer rooming-in of family members not only for pediatric patients.

At the same time, medical staff can be supported by family members, who in turn can be informed and taught about the disease and their role as caregivers before taking over responsibility at home. Additionally, within the center, formal (e.g., case conferences) interprofessional working structures can be facilitated by working in local teams, which are still quite uncommon in Germany [39,40]. Integrating nursing, coordination and patient information services in the center’s concept can provide indispensable additional value to patient care in the sense of integrated care [31]. As highly qualified nurses (such as advanced practice nurses) are a predefined part of the EOC staff [41], this is easier to be achieved than in traditional care structures. In addition, nurses can take over many tasks traditionally performed by GPs and thus can substantially relieve the GP shortage by addressing their workload [42]. Furthermore, as nurses often consider primary care as an opportunity to offer holistic care and young doctors prefer to work in (interprofessional) teams, this can contribute to attracting qualified staff.

Thus, based on the results of this study as well as pertaining literature: It is expected that coordination and continuity of care can be efficiently improved by the larger interprofessional structure as such. Without the need for a complex hospital infrastructure, the option of overnight stays adds to securing regional and safe care. In addition, the range of services offered (in the outpatient setting) can be significantly expanded, especially through the option of overnight monitoring.

### 4.2. System and Economic Perspective

In line with the international trend to avoid hospital admissions [25,43], EOC can fill the gap where hospital-level inpatient services are not necessary (or not available), and classical outpatient care does not suffice. This can free hospitals of economically burdensome patients (low case complexity) and improve patient care at the same time, and our models show that such facilities could be significantly more cost-effective compared with treatments in a regular hospital infrastructure [1]. Substitution of a physician with a specialized nurse adds to the cost-efficiency. Overall, the lower physician-per-patient ratio is compensated by a higher nurse-per-patient ratio, due to the smaller ward size. In the underlying concept [2], the size of the EOC unit was set to 15 beds, where the overnight monitoring would need 12 FTE nurses, and the physician workload would equal 1 FTE that could be distributed amongst the collaborating physicians in the IHC, plus out-patient care. In the German setting, however, financing, as well as the legal relation of this intersectoral structure, still requires pilot projects and has not yet made its way into regular care, despite numerous calls for action [21,44].

### 4.3. Regional Structures and Implementation

In line with the German Medical Council’s recommendations of 2014 [22], IHC should always be adapted to regional needs and include (as much as possible) prevailing structures and competencies. Not only to comply with local planning guidelines, but the goal should also be to include local GP and specialist practices in the center. Particularly, partners necessary for optimal diagnostic security, such as radiology (practices) and emergency rooms of regional hospitals (linked by telehealth applications), should be part of the network. Affiliations with the center can be organized in a flexible manner, ranging from full spatial and organizational integration, to satellite practices, to occasional cooperations supported by telehealth. In order to avoid additional fragmentation of care, aligning cooperation and communication processes with short- and long-term nursing facilities as well as social support, seems indispensable. Local pharmacies, therapists as well as services offering patient education and prevention should be involved in the planning process as early as possible.

### 4.4. Limitations

Even if these results originate from the context of a German setting, where a stark intersectoral divide between in- and outpatient care results in suboptimal care of the patient, these results can serve as an orientation for other healthcare settings. Our model was targeted predominantly at non-surgical cases, which would either be treated in medical departments of hospitals or in primary care where resources are insufficient. Therefore, it can complement other models, such as micro-hospitals in the US [7], or outpatient surgery. As evaluated use-cases do not yet exist in the German setting, the concept of IHC and EAV is primarily based on expert opinion, international evidence and literature at this point; it is a concept and no proof thereof. As patients were not included in the interview process, the discussion of patient-orientated care is purely based on the perception of health care professionals. Presently, for EOC, neither financing nor legal aspects of cooperation are defined in the German context. The recent expertise of the government commission for modern and needs-based hospital care suggesting a new hospital level, according to EAV, shows the appeal of the model.

## 5. Conclusions

Various patient groups can benefit from an intermediate structure, such as EOC in a healthcare setting, and can be defined by diagnosis and related criteria. The necessary basic infrastructure resembles that of GP practices, complemented by a monitoring unit, and adapted to the specific range of locally offered services. The range of procedures to be offered in a center is dependent on the regional network and can be grouped into (1) diagnostics (divided into technical, consultant or lab-based), (2) monitoring and assessments, (3) planning and coordination of therapy and (4) therapy itself (invasive procedure, medication, nursing care).

The suggested new care structure at the intersection between in- and outpatient care can provide a solution to rural regions to supplement primary care where hospitals are difficult to sustain. It can provide a more comprehensive, interprofessional and continuous care close to home, especially for the growing geriatric population. Therefore, it is also geared more to patients’ needs than to existing sector-bound structures. At the same time, it can relieve hospitals of misplaced cases, provide attractive work-places for staff and thus address the lack of medical staff.

In the next step, pilots with accompanying evaluations that explore possible ways of integrating the model into the present formal framework, are urgently needed.

## Figures and Tables

**Table 1:** Expert interview partners.

	Outpatient Practice	Hospital	Rural	Urban
General Practitioner	8		4	4
Internal Medicine		2	2	
Internal Medicine	1			1
Cardiologist	1			1
Cardiologist		1		1
Pulmonol./Card		1		1
Oncologist/gastro		1		1
Oncologist/Geriatrician		1		1
Surgeon	1		1	
Dermatologist	1			1
OB Gyn	1	(1) *		1
Neurologist	1			1
ENT	1	(1) *		1
Pediatrician	2		1	1
Pediatrician		1		1
Psychosomatic	1	(1) *		1
Psychiatrist		1		1
Radiologist	1			1
Pain-specialist	1			1
Nurse	2	1	1	2
**Total**	**22**	**9**	**9**	**22**
Healthcare Consultant	1			
CEO of admission ward	1			

* Worked in an inpatient setting up to two years ago.

**Table 2:** Groups of scenarios in acute and chronic conditions suitable for EOC.

Category	Description	Examples
**Acute, potentially** **life-threatening conditions**	not necessarily needing full hospital setting, but intensified monitoring and adjustment	Pneumonia, pyelonephritis, erysipelas, exsiccosis, renal colic
**Crisis intervention**	Pneumonia, pyelonephritis, erysipelas, exsiccosis, renal colic	Panic attacks, counseling domestic violence
**Exacerbation/decompensation** **in chronic diseases**	especially geriatric/multimorbid patients	Known heart-/liver-/kidney insufficiency; asthma, COPD
**Palliative therapy and** **supportive care**	not necessarily needing full hospital setting, but support or interprofessional care	Pain management, chemotherapy-associated side effects, geriatric trauma
**Diagnostic or** **therapeutic procedures**	difficult to implement in outpatient setting or needing cooperation with specialists	Pleura-/ascites-punctures, extensive wound management, catheter management, cardioversion

**Table 3:** Decision categories for the selection of the appropriate care level.

Category	Description
Disease-related criteria	Diseases where a distinct diagnosis can be achieved with available resources Complications that are controllable within the setting of EOC (conversely: Restraint with patients without preconditions and acute potentially threatening symptoms) Conservative therapies that cannot be performed in sufficient quality in regular outpatient care (e.g., i.v.-therapies, monitoring, pain therapy…)
Individual case severity	Patients with known preconditions and controllable/assessable risks (e.g., decompensa-tions, therapy adjustments with required monitoring…). Patients with limited/restrained therapeutic goals (e.g., palliative patients) Patients with complicating comorbidities or frailty
Sociodemographic criteria	Patients with significant need of support (e.g., elderly patients, pregnant women, cogni-tively impaired patients, children or families) Rural areas with long distances to the next hospital Difficult home or social situation: single persons without caregivers, homeless, persons without language or other coping skills
Structural criteria	Available infrastructure that enables necessary diagnostic and therapeutic procedures Available competencies of physicians, nurses, therapists, social workers, etc., in the center as well as in the regional network (including tele-support)

## Data Availability

The data presented in this study are available on request from the corresponding author. The data are not made publicly available due to German language.
